# Development of Molecularly Imprinted Polymer in Porous Film Format for Binding of Phenol and Alkylphenols from Water

**DOI:** 10.3390/ijms15011338

**Published:** 2014-01-20

**Authors:** Andriy O. Gryshchenko, Christina S. Bottaro

**Affiliations:** Department of Chemistry, Memorial University of Newfoundland, St. John’s, NL A1B 3X7, Canada; E-Mail: agrysh@gmail.com

**Keywords:** thin film fabrication, “sandwich” technique, MIP porosity, monomer-template interactions, hydrophobic interactions, styrene, pentaerythritol triacrylate (PETA), methanol/water, phenol adsorption isotherms, imprinting effect

## Abstract

Molecularly imprinted polymers (MIPs) were fabricated on glass slides with a “sandwich” technique giving ~20 μm thick films. Methanol/water as a solvent, and polyethyleneglycol and polyvinylacetate as solvent modifiers, were used to give a porous morphology, which was studied with scanning electron microscopy and gravimetric analysis. Various MIPs were synthesized through non-covalent imprinting with phenol as the template; itaconic acid, 4-vinylpyridine, and styrene as monomers; ethylene glycol dimethacrylate, triethylene glycol dimethacrylate, and pentaerythritol triacrylate (PETA) as cross-linkers. Binding and imprinting properties of the MIPs were evaluated based on phenol adsorption isotherms. Since phenol has only one weakly acidic hydroxyl group and lacks unique structural characteristics necessary for binding specificity, the preparation of selective MIPs was challenging. The recognition of phenol via hydrogen bonding is suppressed in water, while hydrophobic interactions, though promoted, are not specific enough for highly-selective phenol recognition. Nevertheless, the styrene-PETA MIP gave modest imprinting effects, which were higher at lower concentrations (Imprinting Factor (IF) = 1.16 at 0.5 mg·L^−1^). The isotherm was of a Freundlich type over 0.1–40 mg·L^−1^ and there was broad cross-reactivity towards other structurally similar phenols. This shows that phenol MIPs or simple adsorbents can be developed based on styrene for hydrophobic binding, and PETA to form a tighter, hydrophilic network.

## 1. Introduction

Phenol and other phenolics are water pollutants that orignate from various sources, such as oil extraction and treatment, wood and coal pyrolysis, and industrial organic synthesis [1]. Due to its toxicity and abundance, the United States Environmental Protection Agency (US EPA) has placed phenol on their list of Priority Pollutants, which specifies a safe level of 2 mg L^−1^ in drinking water [2] and 0.001 mg L^−1^ in water to be chlorinated [3], for example, in drinking water treatment. Materials for adsorption of phenol and other phenolics are widely used in wastewater clean-up, solid-phase extraction for chromatographic analysis, and sensors. In all these cases, adsorption can be effectively completed with molecularly imprinted polymers (MIPs) [4–6]. A MIP is a synthetic material with template-shaped vacant sites, which can bind molecules of specific structure and/or functionality. MIPs can be synthesized by different types of imprinting approaches (covalent, semi and non-covalent, with sacrificial spacer, and metal ion-mediation) in a variety of formats (monolith, film, powder, beads, a layer grafted onto surfaces) [7].

Many MIPs have been synthesized for a range of phenols: chlorophenols [4,8,9], nitrophenols [5,10,11], dixydroxyphenols [6,12], nonylphenol [13], and bisphenol A [14], though only few of them target phenol and simple alkylphenols. MIPs for phenol have been prepared by non-covalent imprinting approach in the form of crushed monolith [15], a recognition layer immobilized on silica particles [16], as a membrane [17].

To employ MIPs in miniaturized analysis systems [18], sensors [6], and analytical test systems [17,19], they should be fabricated in a film format. This can be done by spin-coating, “sandwiching”, mixing of MIP particles with a polymer binder, and polymerization within a porous structure of another film [20].

In this work, a non-covalent approach was applied to MIP synthesis because of its simplicity and versatility. The core of this approach is formation of a prepolymerization complex between a template and functional monomer (in solvent) through relatively weak interactions: van der Waals, ionic, hydrogen bonding. After polymerization and template removal, an imprinted site is formed, which can rebind a template with similar non-covalent interactions and in the same configuration that existed in the prepolymerization complex [7]. Here, the polymerization step was completed by “sandwiching” the prepolymerization solution between two glass surfaces to form a thin continuous polymer film.

The goal of this project has been to fabricate and study MIP porous films for phenol using different compositions of monomer, solvent and cross-linker. Complexation between the template and a selected monomer in a certain solvent was confirmed by UV absorbance spectrometry. The morphology of the films was studied with scanning electron microscopy (SEM) and gravimetrical analysis. MIP binding properties were characterized using adsorption isotherms of phenol rebinding from aqueous phenol solutions. The imprinting effects of these MIPs were evaluated based on analysis of the phenol binding isotherms, and cross-reactivity towards other compounds.

## 2. Results and Discussion

### 2.1. MIP Films Prepared by “Sandwich” Technique

**Figure 1.**
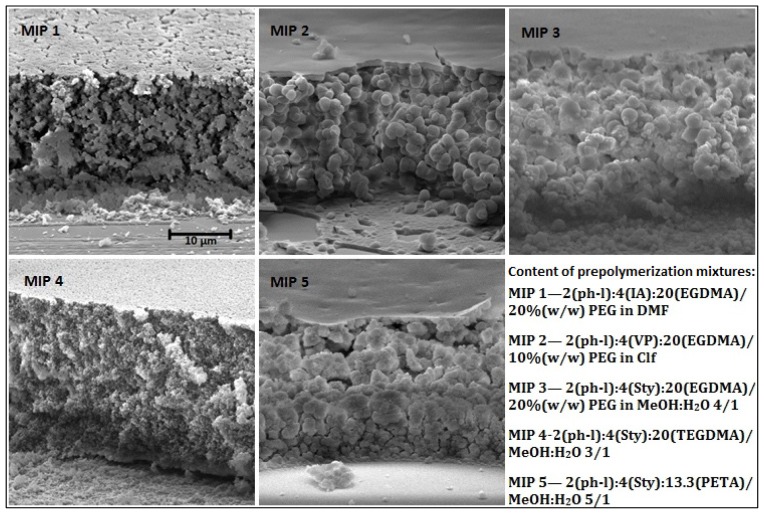
Top-down SEM images of MIP cross-sections.

Different methods for fabrication of MIP films have been discussed elsewhere [7,21,22]. Among them, a spin-coating method has been identified as having the advantage to produce films of controlled and uniform thickness, but it usually requires a low volatility prepolymerization mixture [21]. When more volatile solvents or monomers are used, MIP films can be prepared by a “sandwich” technique, which was applied in this work (described in the Experimental section). MIP films were prepared based on the following components. The monomers selected were: Itaconic acid (IA), 4-vinylpyridine (VP), and styrene (Sty); cross-linkers: ethylene glycol dimethacrylate (EGDMA), triethylene glycol dimethacrylate (TEGDMA), and pentaerythritol triacrylate (PETA). The solvents used were: *N*,*N*-dimethylformamide (DMF), chloroform (CHCl_3_), methanol/water (MeOH:H_2_O); polyvinylacetate (PVA) and polyethyleneglycol (PEG) were also added to the solvent. All fabricated films (MIP 1–5, Figure 1) have been characterized and the morphological details are discussed below. Binding properties will be discussed in sections 2.2, 2.3 and 2.4.

The films, covalently bound to the chemically modified surface of the glass slide, were white and opaque with a visually uniform and even surface. SEM imaging of film cross-sections (Figure 1) showed that the films had a flat surface, which was shaped by the cover glass and the film body had a porous and granular structure. In order to obtain films of this structure, a so called “poor” solvent that causes “reaction-induced phase separation” during polymerization [21] was applied. Linear polymers (PVA and PEG) were added into solvents to aid in the formation of a stable porous/granular MIP (Figure 1); PEG was added to DMF to produce MIP 1 and PVA to CHCl_3_ to give MIP 2 (details on the composition are provided in the Experimental section). PEG and PVA have been used previously as solvent modifiers to render a high porosity to the MIP network, where PEG has been used for membranes [17] and PVA for spin-coated films [21]. For comparison, MIP 1 and 2 formulations were also prepared without these polymeric additives; this resulted in only slightly opaque films of low porosity (Figure S1). These films also shrank with air-drying, and, became very brittle, flaking from the glass slide. This suggests that the porous structure is a significant factor in the mechanical stability of the film. MeOH:H_2_O is a “poor” solvent system itself without any polymeric additives and it was used for production of porous MIP networks previously [19,23]. MeOH:H_2_O was used in preparation of MIPs 3, 4, 5 producing highly porous films. Though the use of PEG to render porosity to MIP 3 films is not necessary, it was added to increase viscosity of the prepolymerization mixture in order to reduce its leakage beyond the cover glass boundary, making films fabrication more facile and reproducible.

The thickness of fabricated films depends mostly on the volume of prepolymerization mixture deposited onto the glass slide and the area the liquid mixture spreads on under the cover glass (25 × 25 mm^2^). The average thickness for all films was estimated to be about 20 μm (Table 1) using SEM. This value is less than the initial thickness calculated (a height, or thickness, of 16 μL liquid enclosed between two 25 × 25 mm^2^ surfaces of glass slide and cover glass should be approximately 26 μm) for the applied volume of the prepolymerization mixture, but the difference is not problematic for the work and can be attributed to the leakage of fluid beyond the cover glass, as well as shrinkage of the polymer network during polymerization. For comparison, mechanical pressure with spring clamps has been applied onto the cover glass to control film thickness during similar fabrication by “sandwiching” [23].

Degree of porosity is an important morphological feature, however, a conventional nitrogen BET analyzer cannot be used to study porosity of these MIP films because each film only weighs a few mg and they are bound to a glass slide. Therefore, quantitative analysis of bulk porosity has been suggested to measure porosity gravimetrically, where specific pore volume (*ν*) is calculated from the volume of absorbed liquid in the film pores normalized to polymer mass. Although it is acknowledged that this method cannot give an indication of pore size distribution, it is easy and does not require any special equipment.

From the data given in Table 1, it can be seen that films have a significant porosity—about 1 mL of pores per gram of polymer network. A comparison of morphologies for MIPs 3, 4 and 5, which use different cross-linkers (section 2.3), shows that the lowest porosity was observed for MIP 4 with TEGDMA. This reflects what is observed in the SEM images (Figure 1) that shows a dense packing of small granules for the TEGDMA MIP. There are at least two potential explanations for this effect. One is that because the composition of the MIPs was based on mole ratios and a fixed volume of solvent (the compositions are described in the experimental section), the MIPs based on TEGDMA had a higher mass concentration in the prepolymerization solution and resulted in a more dense material. The other is related to the length of the spacer in the cross-linker, which in principle allows for formation of a more complexly cross-linked polymeric structure. The higher porosity for MIP 3 (EGDMA) than that for MIP 5 (PETA) is likely due to the trifunctionality of PETA, which should render a higher degree of cross-linking, forming a denser polymer network (section 2.3). The high porosity and the granular film structure suggest that it is possible for the adsorbate (e.g., phenol) to be adsorbed not only at the surface of MIP film but also within film bulk. For this reason, the amount of bound adsorbate (*m*_adsorbate_) was normalized against film mass (*m*_film_) rather than the film surface to get binding capacity (*Q*):

(1) $$Q=\frac{m_{adsorbate}}{m_{film}},mg\cdot g^{-1}$$

### 2.2. Choice of Monomer and Solvent

In development of these phenol MIPs, it was decided to use phenol as the template rather than a pseudo-template for simplicity. In future work, the use of an alkylated phenol or other monoaromatic species as the template would be useful. IA, VP, and Sty were chosen as monomers based on their ability to interact with phenol in different ways, such as hydrogen bonding and π-π interactions. In the choice of solvent other than the condition that it be a relatively “poor” solvent for the polymer components, solvents that would not significantly disrupt the template-monomer interactions in prepolymerization mixture were also considered [24].

**Figure 2.**
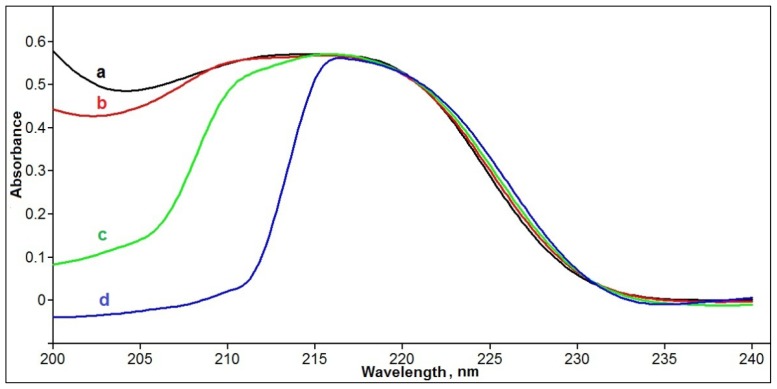
Effect of styrene on E_2_-band of phenol in MeOH:H_2_O (4:1); phenol concentration: 0.1 mM; styrene concentrations: (**a**) 0; (**b**) 0.1; (**c**) 0.2; (**d**) 0.4 mM. Reference cuvette contains the same concentration of styrene but no phenol.

Computational studies carried during the development of phenol MIP membranes have shown that anion of itaconic acid (IA) binds phenolic hydroxyl via hydrogen bonding [17]. In that work, DMF was used as the solvent, probably due to its ability to act as a proton acceptor and, thereby, facilitate itaconic acid dissociation. Thus, the IA/DMF pair was also used in this work. The other pairing of VP and CHCl_3_ was based on the capacity for hydrogen bonding between the basic nitrogen of VP and phenolic hydroxyl, which has been observed in non-polar solvent systems such as CHCl_3_ by various techniques (NMR, IR) [15]. Between styrene and phenol, hydrophobic interactions including π–π stacking can be important. A solvent to promote interactions of this kind can be highly polar and protic like the MeOH:H_2_O mixture. UV absorbance spectrometry is a common technique to study monomer-template interactions by hydrogen bonding [25]. It can be applied to study the hydrophobic interactions in protic solvents, where NMR and IR spectroscopy are not applicable. The complexation of phenol and styrene can be concluded from changes in phenol spectrum (E_2_-band) upon addition of styrene, which were observed for very dilute phenol solutions (Figure 2).

#### 2.2.1. Phenol Binding Studies in Water for MIPs Prepared on Selected Monomers and Solvents

MIP films based on IA, VP, and Sty (MIP 1, 2, 3 in Table 4) were prepared and tested in phenol rebinding from aqueous solutions. For this study, the concentrations used are described as having moderate phenol concentrations (10 and 15 mg L^−1^) and high phenol concentrations (100 and 300 mg L^−1^). At each concentration, the imprinting factor (*IF*) was calculated (Table 2). The *IF* characterizes MIP binding capacity (*Q*_MIP_) over that for non-imprinted polymer (*Q*_NIP_), and it is the simplest estimation of imprinting effect.

(2) $$IF=\frac{Q_{\text{MIP}}}{Q_{\text{NIP}}}$$

For MIP 1 (IA/DMF), IFs at moderate concentrations (10 and 15 mg L^−1^) are slightly higher than those at high concentrations (100 and 300 mg L^−1^). This suggests the presence of higher energy MIP binding sites, which are occupied at low phenol concentrations. However, efficiency of the MIP over the NIP is very modest; and may be because recognition of phenol through hydrogen bonding is suppressed in the aqueous environment. For similar reasons, in case of MIP 2 (VP/CHCl_3_) and the corresponding NIP, the binding capacities are about the same for all phenol concentration range taking the variability into account (*IF* ≈ 1.0). A further factor at play in this system is the mismatch between the highly hydrophobic solvent in prepolymerization mixture (CHCl_3_) and the highly polar and protic water as environment for binding. It has been noted previously that the imprinting effect is more pronounced when the solvent used during the formation of prepolymerization complex has similar properties to the solvent for rebinding [26]. The MeOH/H_2_O solvent system, used for MIP 3 based on styrene, is probably the closest solvent to the water from which phenol rebinding takes place. Although, the hydrophobic interactions between styrene and phenol should be strong in this solvent, it seems that the non-selective hydrophobic interactions dominate over selective interactions associated with imprinted cavities. It was observed that only the IF for the higher phenol concentrations is higher than unity, and only marginally so.

Generally, it is a challenging task to prepare effective MIPs for phenol because it is a small molecule without many special features in terms of shape and functionality. Thus far MIPs for binding phenol from water with only modest imprinting effect (here the imprinting factors were estimated based on MIP and NIP binding behavior at high adsorbate concentrations) have been prepared, for example, ca 1.25 [17], or even less than unity in the case of nonylphenol MIP [13]. Phenol has only one hydroxyl group, therefore, it can be retained in a binding site only by one hydrogen bond. In contrast to phenol, MIPs with higher imprinting factors have been prepared for 2,4-dichlorophenol (2.1) [9], 2,4-dinitrophenol (2.2) [10], bisphenol A [14], hydroquinone (2.2) [12]. These species used as templates have more specific shape and at least two functional groups available for bonding (e.g., two –NO_2_ and one –OH in dinitrophenol). Furthermore, the hydroxyl protons for chloro– and nitrophenol are more acidic than the proton in phenol, which can yield stronger hydrogen bonding with proton accepting monomers. All these factors make the prepolymerization complex more stable, which results in more selective binding sites in the final MIP network, and, consequently, a stronger imprinting effect.

Although, these MIPs did not give satisfactory imprinting effects, other factors with a potential to influence the selectivity of the MIPs, such as the effect of cross-linker are of interest and are useful to study. Based on the somewhat promising results for the imprinting factors, the styrene/MeOH:H_2_O system was chosen for the study of cross-linkers to improve imprinting effect towards phenol; this system also should maximize the hydrophobic interactions that dominate in aqueous environments and these systems have not been studied for phenol imprinting previously.

### 2.3. Choice of Cross-Linker

**Figure 3.**
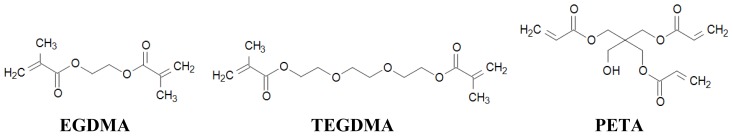
Structures of cross-linkers used.

The cross-linker constitutes most of the MIP by mass (in this work 88%–93% *w*/*w*); therefore, it dictates the structure and tightness of the polymer network [24], and potentially contributes to a significant amount of the non-specific binding [27]. In addition to the styrene MIPs with EDGMA, styrene based MIPs were prepared with two other cross-linkers, TEGDMA and PETA (Figure 3). TEGDMA has been used for synthesis of a variety of resins and MIP membranes [17], and has a long flexible glycol chain. Due to its hydroxyl group, PETA has been used for preparation of hydrophilic MIPs [27]. Also, as a trifunctional cross-linker it is expected to produce a greater degree of cross-linking and tighter polymer network. These cross-linkers were dissolved in MeOH/H_2_O with the highest possible water content that can still produce homogeneous prepolymerization mixtures. It is believed that higher water content in the solvent allows for stronger phenol-styrene interaction in prepolymerization complex. PETA is soluble in 5:1 MeOH:H_2_O in contrast to a widely used trifunctional cross-linker trimethylolpropane trimethacrylate (TRIM), which cannot be dissolved in such polar solvent systems, even acetonitrile. TEGDMA tolerates the highest amount of water, and is soluble in 3:1 MeOH:H_2_O (the composition for EGDMA is 4:1); better TEGDMA solubility is due to a higher number of ethereal oxygen in TEGDMA than EGDMA.

**Figure 4.**
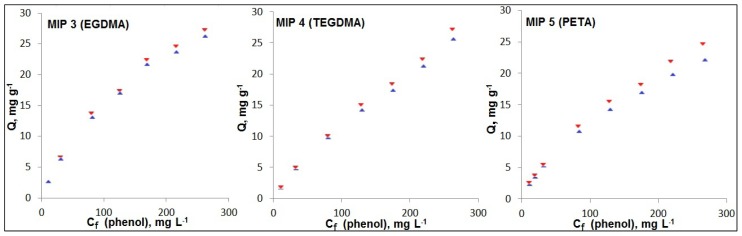
Binding isotherms for Sty-based MIP/NIP on different cross-linkers: EGDMA, TEGDMA, and PETA from 15 to 300 mg L^−1^ phenol concentrations (initial); 
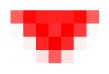
—MIP, 
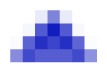
—NIP. Note: *C*_f_—phenol concentration at binding equilibrium.

For MIP 3 (EGDMA), the IF rises from 1.00 at 15 mg L^−1^ to 1.04 at 300 mg L^−1^ (Figure 4 with data in Table S1). In case of MIP 4 (TEGDMA), IF increases from 1.04 to 1.06, within the studied concentration range. For MIP 5 (PETA) IF improves from 1.04 at 40 mg L^−1^ to 1.12 at 300 mg L^−1^; below 40 mg L^−1^ there is a slight increase in IFs as the phenol concentration diminishes (see next section 2.4.1). Comparison of IFs in the region 150–300 mg L^−1^, where the IFs for each MIP exhibit little variation over the range of phenol concentrations, shows that the highest IFs are observed for MIP 5 (PETA). It can be explained by the tighter and more rigid structure of binding sites, which better fit phenol as a small molecule. An average IF for TEGDMA-MIP is slightly higher than that for EGDMA-MIP, probably due to higher water content (see above) in the solvent combined with with tighter structure of the TEGDMA-MIP (MIP 4 section 2.1). Generally, the extent of non-specific binding by an MIP can be assessed based on binding capacity for its NIP. Comparison of the NIP binding capacities (Table S1) for all studied phenol concentrations demonstrates that non-specific hydrophobic binding towards phenol is lower for MIPs on TEGDMA and especially on PETA.

Another feature of the MIP films based on PETA and TEGDMA is that they were easily wetted with water; whereas the Sty-MIP film with EGDMA had to be conditioned in acetonitrile:water (1:1). This dependence of wetting and non-specific binding on the cross-linker type can be explained by the higher hydrophilicities of TEGDMA and PETA when compared to EGDMA; TEGDMA has long hydrophilic glycol chain, and PETA possesses a hydroxyl group and lower carbon content due to acrylic moieties instead of methacrylate. Thus, TEGDMA and PETA can be recommended as cross-linkers for water-compatible MIPs with less non-specific binding in water towards hydrophobic species. In light of the higher imprinting factor and water compatibility, the PETA-MIP was chosen for more detailed study of binding characteristics, which is presented in the next section.

### 2.4. Characterization of Styrene/PETA MIP (MIP 5)

#### 2.4.1. Binding Properties Study

It was mentioned previously (section 2.3) that there is a breakpoint in the isotherm for MIP 5 around 40 mg L^−1^, where the MIP 5 isotherm begins to diverge from the NIP isotherm with rising IFs towards both high and low phenol concentrations (Tables S1 and S2). It is known that lack of uniformity in MIP binding behavior can occur because the MIP shows different binding site distributions depending on adsorbate concentration range [25,28,29].

**Figure 5.**
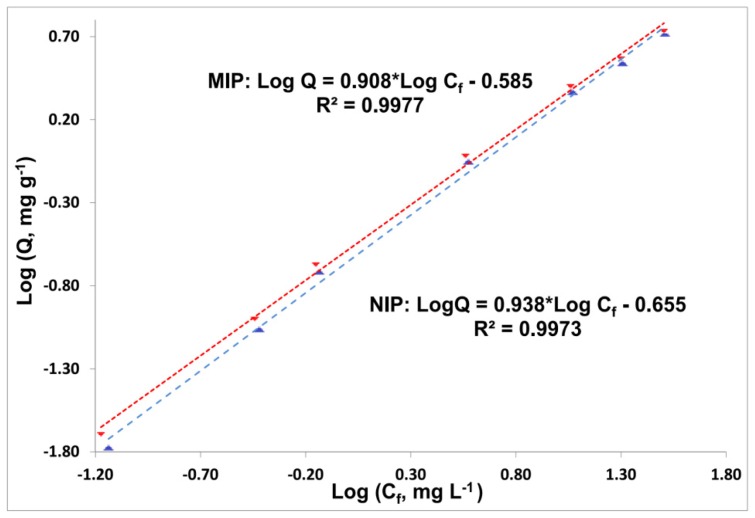
Phenol binding isotherms for 5 MIP and NIP (PETA) in log–log format and fitting to the Freundlich binding model: 
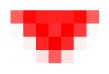
 MIP, 
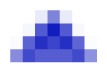
 NIP.

In practice, the concentration of phenol in natural and sewage waters is in the μg L^−1^ to mg L^−1^ range, therefore, it is appropriate to study phenol MIP binding behavior, including binding sites distribution, in a low phenol concentration region. Thus, NIP and MIP 5 isotherms were built from to 0.1 to 40 mg L^−1^ (Figure 5 and Table S2). The imprinting factors, reflecting the efficiency of the MIP over its NIP, showed a steady increase with decreasing phenol concentration (Table S2): IF = 1.04 at 40 mg L^−1^, 1.07 at 25 mg L^−1^, 1.16 at 0.5 mg L^−1^ and 1.20 at 0.1 mg L^−1^. In this concentration window, the MIP and NIP isotherms can be linearized on a logarithmic scale (Figure 5), which means that they are described well by the Freundlich isotherm (FI) binding model. For comparison, linearization with the Langmuir binding model [25,30], which corresponds to unimodal affinity distribution, gives a worse fit with R^2^ values of 0.9474 (MIP) and 0.8608 (NIP) (Figure S2).

According to the FI, the amount of bound adsorbate, expressed as binding capacity (*Q*), depends on free adsorbate concentration (*C*) in a power of *m* as:

(3) $$Q=a\;C^{m}$$

or in a linearized form

(4) Log Q=m Log C+Log a

where *m* and *a* are fitting parameters connected with adsorbent binding properties.

The FI pattern corresponds to the asymptotically decay region of the affinity distribution (Figure S3), which usually takes place within limited interval of adsorbate concentrations, which are usually at low levels [30,31].

The heterogeneity index, *m*, is a value between 0 and 1 that characterizes the “ratio of high-to-low affinity sites”. The lower the *m* value, the higher heterogeneity, meaning a greater proportion of high affinity binding sites in the affinity distribution [31], which is the case for MIP 5 compared to its NIP (Table 3). Based on fitting parameters *m* and *a*, the apparent number of binding sites, *N_K1–K2_*, and apparent weighted average affinity, *K_K1–K2_*, were calculated (formulas in SI) for the range of affinity constants, *K_1_–K_2_*, set by the concentration limits of these experimental isotherms (Table 3). Relative to the NIP, the MIP has higher *N_K1–K2_*, and greater degree of heterogeneity (*m*) resulting in slightly higher average affinity *K_K1–K2_*, which all prove a modest imprinting effect [31].

#### 2.4.2. Cross-Reactivity Study

**Figure 6.**
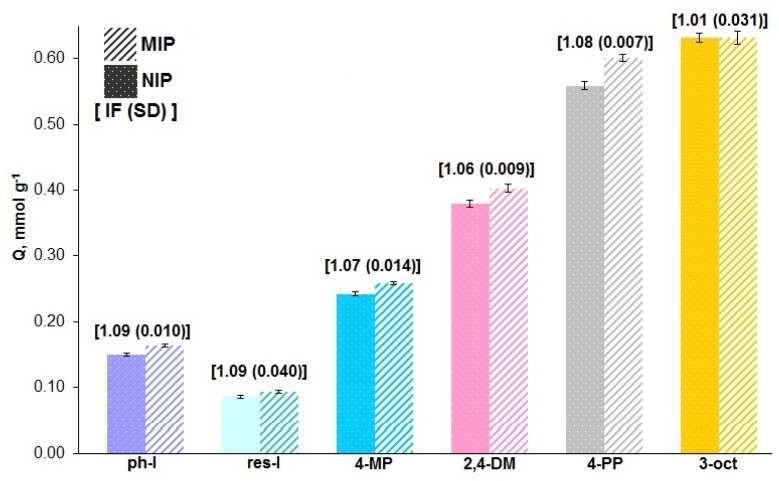
Cross-reactivity of MIP 5 (PETA) towards other phenols and 3-octanone, *C*_i_(adsorbate) = 1.594 mM. Note: IF is an average of ratios of *Q*_MIP_ to *Q*_NIP_ (*n* = 4, SD in parenthesis); *Q*_MIP(NIP)_ is an average corresponding to different batches (*n* = 4, SD error bars).

Cross-reactivity of the PETA MIP was evaluated based on a comparison between binding for phenol (ph-l) and structurally-related phenols: resorcinol (res-l); 4-methylphenol (4-MP); 2,4-dimethylphenol (2,4-DM), 4-propylphenol (4-PP), and 3-octanone (3-oct). Figure 6 shows that this MIP has comparable cross-reactivity in terms of IFs towards other phenols, which are different from phenol by one or two substituents on the aromatic ring. This is consistent with the low specificity associated with binding by hydrophobic interactions, which has been discussed previously [32]. Both MIP and NIP binding capacities rise with adsorbate hydrophobicity. For example, the octanol—Water partition coefficients (log *P*) e.g., 1.48, 1.97, 2.35 for phenol, 4-methylphenol, and 2,4-dimethylphenol, respectively [33], increase with alkyl substitution which parallels the trend in binding capacities. Virtually no difference is observed for MIP and NIP binding capacities of 3-octanone, which is non-aromatic in nature and significantly different from phenol structurally. Thus, the modestly higher uptake of phenols is likely due to some molecular recognition capability and their aromatic nature. Such wide selectivity of molecular imprinting by hydrophobic interactions can be advantageous for separation of a whole class of phenols including alkylphenols, which are all of environmental importance.

## 3. Experimental Section

### 3.1. Materials

All chemicals were purchaced from Sigma-Aldrich (Oakville, ON, Canada) unless otherwise indicated. Phenol, resorcinol, 4-methylphenol, 4-propylphenol, 2,2-dimethoxy-2-phenylacetophenone (DMPA), itaconic acid and styrene were or 99% purity. 2,4-Dimethylphenol, 3-(trimethoxysilyl)propyl methacrylate, ethylene glycol dimethacrylate, 3-octanone, 1-decanol were 98% pure. Triethylene glycol dimethacrylate and 4-vinylpyridine were at 95%; pentaerythritol triacrylate was technical grade. Polyethyleneglycol was M*w* 20,000, and polyvinylacetate was M*w* 100,000). *N*,*N*-Dimethylformamide (DMF) was of ACS regent (Sigma-Aldrich, Oakville, ON, Canada) grade with <0.005% water, phenol-2,3,4,5,6-d5, was 98% deuterated. Chloroform (stabilized with amylenes), methanol, acetic acid, hydrochloric acid (37% *w*/*w*) were of ACS reagent grade and were purchased from ACP Chemicals (Montreal, QC, Canada). Hydrophilic polypropylene membrane GHP-200, 0.2 μm were from Pall Corporation (Mississauga, ON, Canada); micro cover glasses 25 × 25 mm^2^ and plain glass microscope slides 75 × 25 mm^2^ were from Fisher Scientific (Ottawa, ON, Canada). All solutions were prepared with 18.2 MΩ·cm water from Barnstead NanoPure Diamond (18 MΩ) water purification system (Barnstead Nanopure Water Systems, Lake Balboa, CA, USA).

### 3.2. Study of Phenol-Styrene Interactions by UV Absorbance Spectrometry

A series of solutions with fixed phenol (0.1 mM) and increasing styrene concentrations (0.1; 0.2; 0.4 mM) were prepared in MeOH/H_2_O (4:1). Absorbance spectra of these solutions were measured with a Thermo Scientific Evolution 600 UV-Vis Spectrophotometer (Thermo Scientific, Ottawa, ON, Canada) against reference solutions with the same concentrations of styrene (0.1; 0.2; 0.4 mM) but without phenol.

### 3.3. Fabrication of MIP Films by “Sandwich” Technique

In a vial, phenol (the template), monomer, and cross-linker were mixed in 1:2:10 molar ratio except MIP 5 where PETA (a trifunctional cross-linker) was used in a lower molar ratio (1:2:6.67) to maintain the same ratio of vinyl groups in reaction mixture. Then, the UV-initiator (DMPA) and solvent (1 mL for all mixtures) were added according to Table 4.

**Figure 7.**
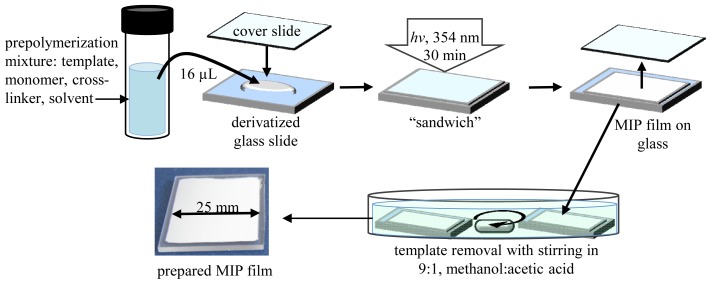
General scheme of fabrication of MIP films by “sandwich” technique.

The mixture was sonicated for 5 min under nitrogen. Glass slides were cut in 3 pieces 25 × 25 mm^2^, soaked in MeOH:HCl_37%_ (1:1) and silanized with 3-(trimethoxysilyl) propylmethacrylate [19]. A scheme of the procedure is presented in Figure 7. A 16-μL aliquot of prepolymerization mixture was delivered with a displacement pipette onto the glass slide and quickly covered with a cover glass before illumination with the UV light (UVG-54, 6W, 254 nm, (VWR, Mississauga, ON, Canada)) for 30 min. The cover glass was removed immediately following the polymerization. Template, the labile linear polymers and unreacted species were removed in methanol/acetic acid (9:1 *v*/*v*) with stirring. The total time of the washing was 19 h 30 min using three fresh portions of the mixture. Polymerization at the slide edges (~2–3 mm in width) was effected by oxygen in air and solvent evaporation; these parts were trimmed with a razor to yield a film of completely homogeneous texture, care was taken not to scratch the glass. Finally, the films were stirred in methanol for 30 min and air dried. The non-imprinted polymer (NIP) was prepared simultaneously without phenol using the same procedure.

### 3.4. Gravimetrical Analysis of Porosity

The volume of liquid absorbed into the porous structure of MIP film bound to glass was determined using the mass difference between the MIP film soaked with 1-decanol (*m_l_*) at room temperature and the initial dry MIP film accounting the density of 1-decanol (*ρ_l_*, e.g., 0.829 g mL^−1^ at 25 °C). Normalization of this volume to film mass (*m*)gives specific pore volume (*υ*):

(5) $$v=\frac{m_{l}}{\rho_{l}\;m}$$

The MIP film mass (*m*) was obtained by subtraction of glass slide mass before polymerization from mass of the slide with bound MIP (as in Section 3.6). Mettler Toledo XS 105 (Mettler Toledo, Mississauga, ON, Canada) analytical balances with accuracy to 0.01 mg was used for mass measurements. 1-Decanol was chosen because of its low volatility and non-hydroscopic properties to ensure minimal uncontrolled mass change for the soaked films. A MIP film/slide was placed film-down on several layers of GHP-200 membrane (with smooth surface), which were moistened with 1-decanol, and the slide was slightly pressed onto the membrane to completely soak the porous film; this was assessed visually. Excess 1-decanol was removed by gentle contact with a section of membrane. Porosity values for four films of the same kind were averaged. No statistically significant difference between the specific pore volumes of MIPs and their blanks was observed.

### 3.5. SEM Imaging and Thickness Measurements

An edge of the MIP films on glass was removed using a razor and the film was coated with sputtered gold. An FEI Quanta 650F field emission scanning electron microscope (FEI, Hillsboro, OR, USA) was used for imaging of the MIP film section with secondary electrons at a 70° tilt and 10 kV acceleration potential. A thickness of film (*H*) was estimated based on average height of seeming 90° section measured on SEM image (*H′*) and the tilt angle:

(6) $$H=\frac{H^{\prime }}{\text{sin }70{}^{\circ}}$$

Thickness values for three films of the same kind were averaged.

### 3.6. Adsorbate Binding Studies

Adsorption by the MIP was completed until binding equilibrium was reached. For calculation of binding capacity for adsorbate (*i.e.*, *Q* as expressed in Equation (1)), the amount of bound adsorbate (mg) was found based on a difference between an initial adsorbate concentration (*C_i_*) and a concentration at equilibrium (*C_f_*). The mass of MIP film (*m*_film_, ~4 mg) was measured as was described in Section 3.4 and expressed as follows.

(7) $$Q=\frac{V(C_{i}-C_{f})}{m_{\text{film}}}$$

MIPs 2 and 3 together with corresponding NIPs were firstly pre-wetted with acetonitrile:water (1:1) while other films were used directly. A 50-mL beaker with a MIP film on a glass slide and aqueous adsorbate solution was sealed with paraffin film and was shaken at 150 rpm (rotations per minute) at 20.0 °C for 4 h 30 min in an Innova 4230 Incubator Shaker (New Brunswick Scientific, Enfield, CT, USA). The volume of the adsorbate solution (*V*) was proportional to the mass of MIP film. This ratio was chosen to be 0.44 mL mg^−1^ for resorcinol and 3-octanone and 0.714 mL mg^−1^ for other compounds to have an optimal difference *C_i_* *–C_f_* for determination of *Q*.

Quantitation of the free adsorbate concentrations was completed with an Agilent 1100 Series LC-MS equipped with a diode array detector (Agilent Technologies Canada Inc., Mississauga, ON, Canada). Chromatographic parameters were the following: Phenomenex Synergi Fusion-RP column (150 × 4.6 mm; particle size 4 μm, Phenomenex, Torrance, CA, USA); isocratic elution with acetonitrile/water mobile phase (Table 5); injection volume varied from 100 to 10 μL depending on analyte concentration. Calibration solutions were the same as used for rebinding experiments and were prepared by their dilution to bracket measured concentrations.

More sensitive quantification of solutions with phenol concentration around 0.1 mg L^−1^ was achieved with mass spectrometry using APCI (atmospheric pressure chemical ionization), and internal standardization with phenol-d5. A small correction for phenol template bleeding from the MIP was applied in determination of the adsorption capacities and the imprinting effect using the concentration of phenol extracted with pure water. The detector was operated in standard settings, fragmentation voltage 50 V, negative mode with intensities acquired for ions at *m*/*z* 93 and 98 (C_6_H_5_O^−^ and C_6_D_5_O^−^), isocratic mobile phase MeOH/pure H_2_O (70/30 *v*/*v*) at 1.2 mL min^−1^, injection volume 100 μL.

## 4. Conclusions

The “sandwich” technique can be used for simple fabrication of ~20 μm thick MIP films with a smooth surface from a wide variety of prepolymerization mixtures. These films can be rendered porous by use of “poor” solvents such as methanol-water mixtures or dimethylformamide and chloroform with addition of linear polymers. The fabricated MIP films are suitable for use as recognition layers in sensors and as a phase for microextraction e.g. in microfluidics given their reproducible and consistent porous morphology. Also, the described “sandwich” technique is recommended as a format for screening of MIP formulations in MIP synthesis because of convenience of handling MIPs bound to glass slides, labor and material savings, and applicability to a wide variety of MIP formulations.

MIP films were prepared based on various monomers to bind phenol through hydrogen binding (itaconic acid, 4-vinylpyridine) and hydrophobic interactions (styrene). The binding properties and selectivity of the MIPs were characterized based on phenol adsorption capacities from water solutions studied at equilibrium. It is challenging to develop high affinity MIPs with high selectivity for phenol due to phenol’s small size, simple shape and only one weakly acidic hydroxyl. In aqueous environments, selective binding of phenol via hydrogen bonding is suppressed, while binding with MIP made with styrene is not significantly specific because binding is mainly through hydrophobic and π–π interactions associated with the aromatic structures of the monomer and phenols. The denser more rigid structure of styrene-based MIPs achieved with trifunctional PETA is beneficial for phenol recognition. Also, the high hydrophilicity of this cross-linker makes MIP films water-compatible and reduces the non-specific binding. Binding isotherms for phenol uptake at low concentrations by styrene and PETA based MIPs showed that the imprinting factors are higher for lower phenol concentrations. The MIP and NIP isotherms follow the Freundlich binding model. The analysis of the isotherms conveys moderately better binding parameters for the MIP over its NIP, and a limited number of selective imprinted sites that are mostly occupied at low phenol concentrations. Although, the selectivity is not exceptional, MIPs with this composition can be used for the binding other structurally similar phenols.

## 5. Future Prospects

The authors believe that this work is a beginning step for the development of more efficient systems for binding of phenol and alkylphenols in water, and the basic components for these MIPs or non-imprinted simple adsorbent phases can be styrene, PETA, and methanol/water as monomer, cross-linker and solvent, respectively. A number of approaches can be used in efforst to improve the imprinting effect, for example, use of higher monomer and template content, polymerization at lower temperatures [31], and addition of a proton accepting monomer with styrene to improve hydrogen bonding [7]. Studies of phenol rebinding over shorter intervals, *i.e.*, before adsorption equilibrium is reached, may also yield better imprinting factors.

## Supporting Information

In this section more information is presented on MIP film morphology (Figure S1), the data for isotherm figures (Tables S1 and S2), fitting experimental isotherms to Langmuir binding model (Figure S2), Freundlich isotherm affinity distribution (Figure S3), and formulas to calculate binding parameters according to Freundlich isotherm binding model.

Calculation of binding parameters and affinity distribution based on Freundlich Isotherm fitting parameters [31].

(S1) $$N_{K1-K2}=a(1-m^{2})(K_{1}^{-m}-K_{2}^{-m})/M,mmol\cdot g^{-1}$$

(S2) $$K_{K1-K2}=\left(\frac{m}{m-1}\right)\left(\frac{K_{1}^{1-m}-K_{2}^{1-m}}{K_{1}^{-m}-K_{2}^{-m}}\right),L\cdot mg^{-1}$$

(S3) $$N(K)=\left[2.303am(1-m^{2})e^{-2.303mLogK}\right]/M,mmol\cdot g^{-1}$$

*m* and *a*—Freundlich Isotherm fitting parameters; *K*—affinity constant at phenol concentration (*C*): *K=1/C*, L·mg^−1^; *M*—phenol molar mass; *N_K1–K2_*—apparent number of binding sites; *K_K1–K2_*—apparent weighted average affinity, *N*(*K*)—an affinity distribution function: a relation between the number of binding sites (*N*) with a certain affinity constant and this constant value (*K*).

Figure S1. Morphology of MIP films prepared without polyethylene glycol (PEG) and polyvinylacetate (PVA). MIP 1 (no PEG): (**A**) SEM image, (**a**) photo; composition of prepolymerization mixture: 2(ph-l):4(IA):20(EGDMA)/pure DMF; MIP 2 (no PVA): (**B**) SEM image, (**b**) photo; composition of prepolymerization mixture: 2(ph-l):4(VP):20(EGDMA)/pure CHCl_3_.

**Table S1.** Data for binding isotherms for Sty-based MIP/NIP on different cross-linkers for Figure 4.

*C* _i_(phenol), mg·L ^−1^	15	40	100	150	200	250	300
**MIP 3 (EGDMA)**							

***Q* (MIP), mg**· **g** ^−^ **^1^**	2.60	6.30	13.0	17.0	21.7	23.7	26.2
***Q* (NIP), mg**· **g** ^−^ **^1^**	2.60	6.51	13.7	17.3	22.4	24.6	27.2
**IF**	**1.00**	**1.03**	**1.05**	**1.02**	**1.03**	**1.04**	**1.04**

**MIP 4 (TEGDMA)**							

***Q* (MIP), mg**· **g** ^−^ **^1^**	1.79	4.91	10.1	14.9	18.4	22.3	27.1
***Q* (NIP), mg**· **g** ^−^ **^1^**	1.72	4.76	9.7	14.2	17.4	21.2	25.6
**IF**	**1.04**	**1.03**	**1.04**	**1.05**	**1.06**	**1.05**	**1.06**

**MIP 5 (PETA)**							

***Q* (MIP), mg**· **g** ^−^ **^1^**	2.52	5.41	11.5	15.4	18.2	21.9	24.6
***Q* (NIP), mg**· **g** ^−^ **^1^**	2.31	5.20	10.7	14.2	16.9	19.8	22.1
***IF***	**1.09**	**1.04**	**1.07**	**1.09**	**1.08**	**1.11**	**1.12**

**Table S2.** Initial data for binding isotherms for MIP/NIP 5 (PETA) at low phenol concentrations (0.1–40 mg L^−1^) for Figure 5.

*C* _i_(phenol), mg·L ^−1^	0.1	0.5	1	5	15	25	40
***Q* (NIP), mg**· **g** ^−^ **^1^**	0.0168 (0.0043)	0.0865 (0.0008)	0.192 (0.009)	0.882 (0.007)	2.31 (0.011)	3.45 (0.13)	5.20 (0.28)
***Q* (MIP), mg**· **g** ^−^ **^1^**	0.0202 (0.0051)	0.1004 (0.0011)	0.212 (0.0001)	0.957 (0.025)	2.52 (0.020)	3.70 (0.11)	5.41 (0.32)
***IF***	**1.20 (0.023)**	**1.16 (0.016)**	**1.11 (0.0044)**	**1.09 (0.0200)**	**1.09 (0.0037)**	**1.07 (0.0060)**	**1.04 (0.0080)**

**N**ote: SD (standard deviation) in parenthesis: *n* = 2 for 1, 5, 15 mg L^−1^; *n* = 3 for 25, 40 and *n* = 4 for 0.1, 0.5 mg L^−1^ points.

Figure S2.Phenol binding isotherms for 5 MIP and corresponding NIP (PETA) in Q/C*_f_*-Q format and Langmuir binding model fits [25] to them: ( 
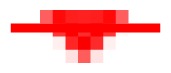
 MIP, 
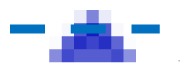
 NIP).Note: Q—binding capacity, C*_f_*—phenol concentration at adsorption equilibrium.

Figure S3.Affinity distributions corresponding to Freundlich Isotherm binding model [31] for MIP 5 on PETA ( 
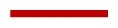
) and corresponding NIP ( 
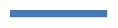
) calculated based on binding parameters (Formula S3).

## Figures and Tables

**Table 1.** Thickness (by SEM) and porosity (gravimetrical method) of films fabricated by the “sandwich” technique. (SD—standard deviation).

Characteristic determined	MIP				

	1	2	3	4	5
H (SD, *n* = 3), μm	24.1 (2.5)	23.4 (3.5)	18.5 (3.1)	21.5 (5.2)	20.6 (2.5)
*ν* (SD, *n* = 4), mL·g^−1^	1.22 (0.04)	1.08 (0.08)	1.46 (0.05)	0.76 (0.07)	0.91 (0.04)

**Table 2.** Imprinting factors for MIP formulations prepared on different monomers.

MIP (composition)	*C_i_* (phenol), mg L^−1^			
	10	15	100	300
	*IF* (SD, *n* = 2)			
MIP 1 (IA/DMF)	1.04 (0.006)	1.04 (0.013)	1.01 (0.007)	0.99 (0.030)
MIP 2 (VP/CHCl_3_)	1.02 (0.013)	0.96 (0.170)	0.99 (0.022)	1.00 (0.015)
MIP 3 (Sty/(MeOH:H_2_O)]	1.01 (0.020)	1.00 (0.016)	1.05 (0.025)	1.04 (0.010)

Note: C_i_—phenol concentration before the binding studies; SD-standard deviation.

**Table 3.** Parameters for fitting to Freundlich isotherm model and calculated binding parameters.

Adsorbent	*R*^2^	*a*, mg g^−1^ (mg L^−1^)^−m^	*m*	*N_K_*_1–_*_K_*_2_, mmol g^−1^	*K_K_*_1–_*_K_*_2_, L mg^−1^
MIP 5	0.9977	0.260 (0.012)	0.908 (0.020)	0.0112 (0.0018)	0.237 (0.011)
NIP 5	0.9973	0.221 (0.012)	0.938 (0.022)	0.0073 (0.0022)	0.221 (0.010)

Notes: *K*_1_ = 0.0313; *K*_2_ = 14.9 (L mg^−1^); SD for log a and m values were calculated in Excel with LINEAST function and on their base SD for a, *N_K1-K2_*, *K_K1-K2_* were calculated by the uncertainty propagation and presented in parenthesis.

**Table 4.** Composition of MIP prepolymerization mixtures.

Polymer component	MIP 1	MIP 2	MIP 3	MIP 4	MIP 5
template	phenol 0.4 mmol (37.6 mg)				

monomer	IA 0.8 mmol (104 mg)	VP 0.8 mmol (85.4 μL)	Sty 0.8 mmol (92.0 μL)	Sty 0.8 mmol (92.0 μL)	Sty 0.8 mmol (92.0 μL)
cross-linker	EGDMA 4 mmol 755 μL	EGDMA 4 mmol 755 μL	EGDMA 4 mmol 755 μL	TEGDMA 4 mmol 1049 μL	PETA 2.67 mmol 674 μL

photoinitiator	DMPA 0.06 mmol 15.4 mg				

solvent (1000 μL)	15% (*w*/*w*) PEG in DMF	10% (*w*/*w*) PVA in CHCl_3_	20% (*w*/*w*) PEG in MeOH:H_2_O 4:1	MeOH:H_2_O 3:1	MeOH:H_2_O 5:1

MIP 1 (no PEG) and MIP 2 (no PVAc) were prepared on pure dimethethylformamide and chloroform (1000 μL) respectively.

**Table 5.** Elution and detection conditions for the chromatographic analysis.

Analyzed species	Mobile phase (*v*/*v*)		Detection wavelength, nm
	CH_3_CN	H_2_O with 5% CH_3_CN (*v*/*v*)	
phenol	55	45	195 *; 216 *; 272
4-methylphenol	55	45	279
resorcinol	35	65	276
2,4-dimethylphenol	65	35	280
4-propylphenol	65	35	278
3-octanone	85	15	279

* detection at 195 and 216 nm was used for solution with low phenol concentration (≤5 mg L^−1^).
