# Supplementary figures and images for: Development of Molecularly Imprinted Polymer in Porous Film Format for Binding of Phenol and Alkylphenols from Water
Source: Int J Mol Sci. 2014 Jan 20;15(1):1338–57. doi: 10.3390/ijms15011338 (PMC3907872; doi:10.3390/ijms15011338)

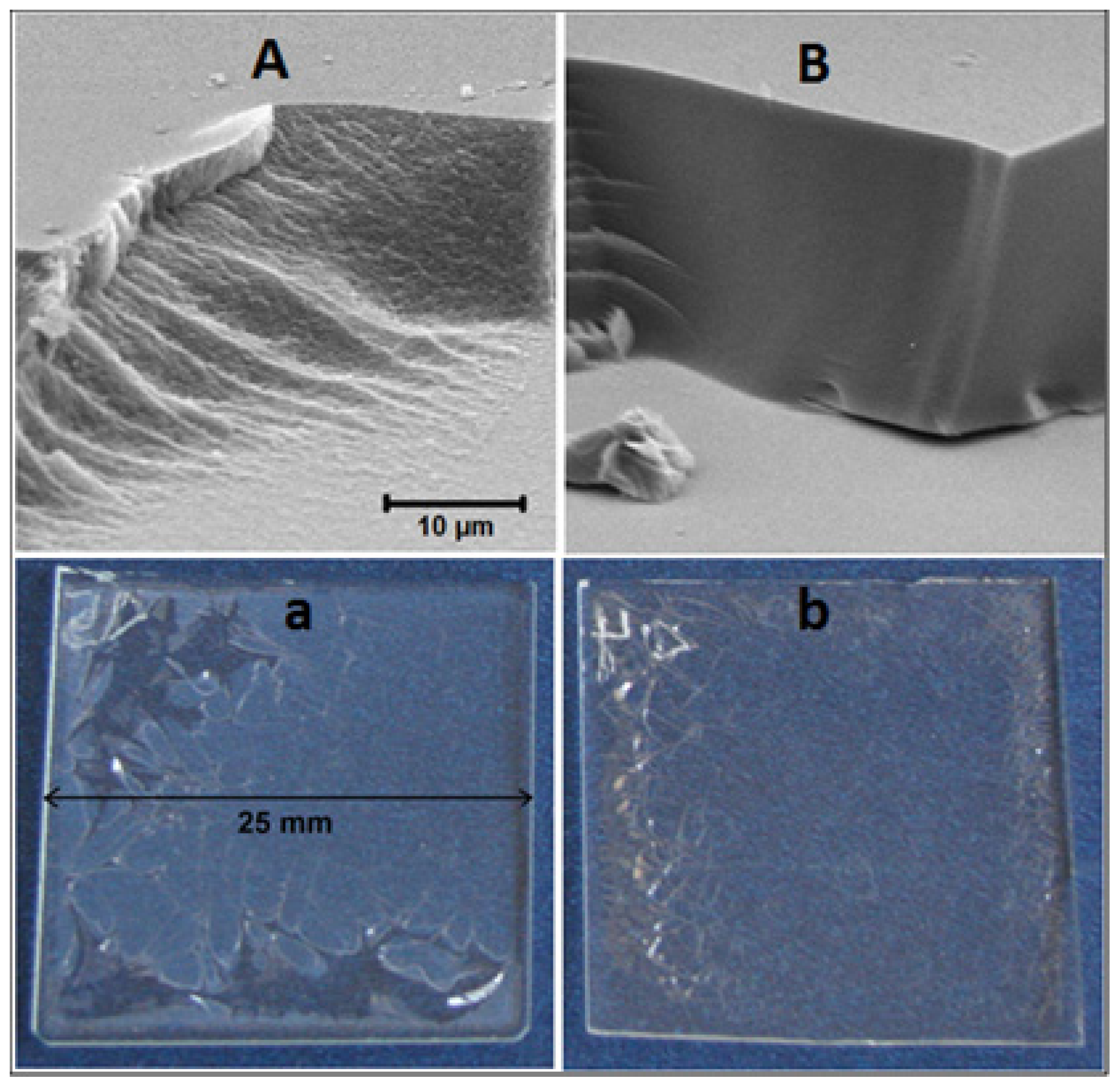

Supplement: Figure S1. — Morphology of MIP films prepared without polyethylene glycol (PEG) and polyvinylacetate (PVA). MIP 1 (no PEG): (A) SEM image, (a) photo; composition of prepolymerization mixture: 2(ph-l):4(IA):20(EGDMA)/pure DMF; MIP 2 (no PVA): (B) SEM image, (b) photo; composition of prepolymerization mixture: 2(ph-l):4(VP):20(EGDMA)/pure CHCl3. [file ijms-15-01338s1.tif]

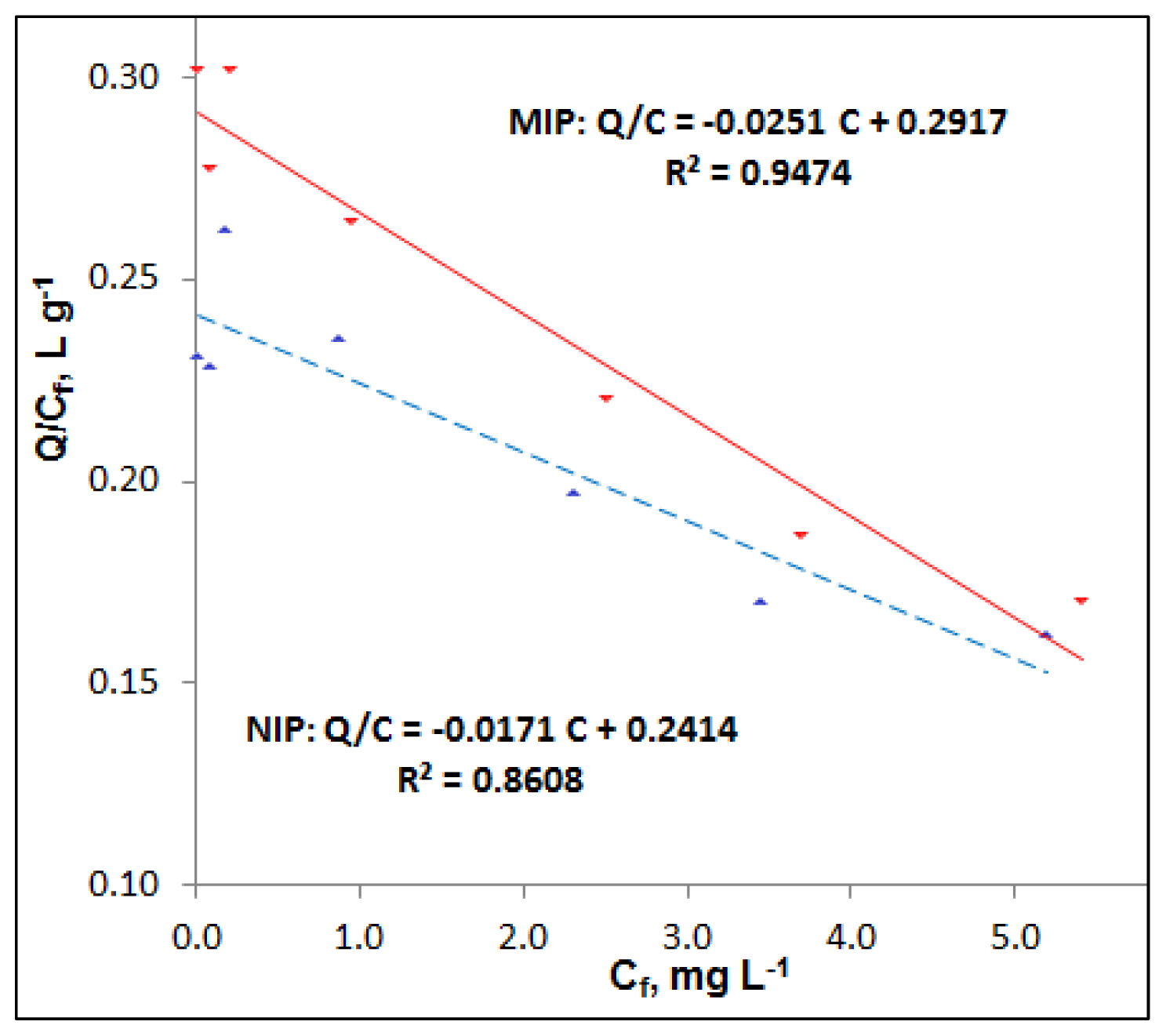

Supplement: Figure S2. — Phenol binding isotherms for 5 MIP and corresponding NIP (PETA) in Q/Cf-Q format and Langmuir binding model fits [25] to them: ( MIP, NIP). Note: Q—binding capacity, Cf—phenol concentration at adsorption equilibrium. [file ijms-15-01338s2.tif]

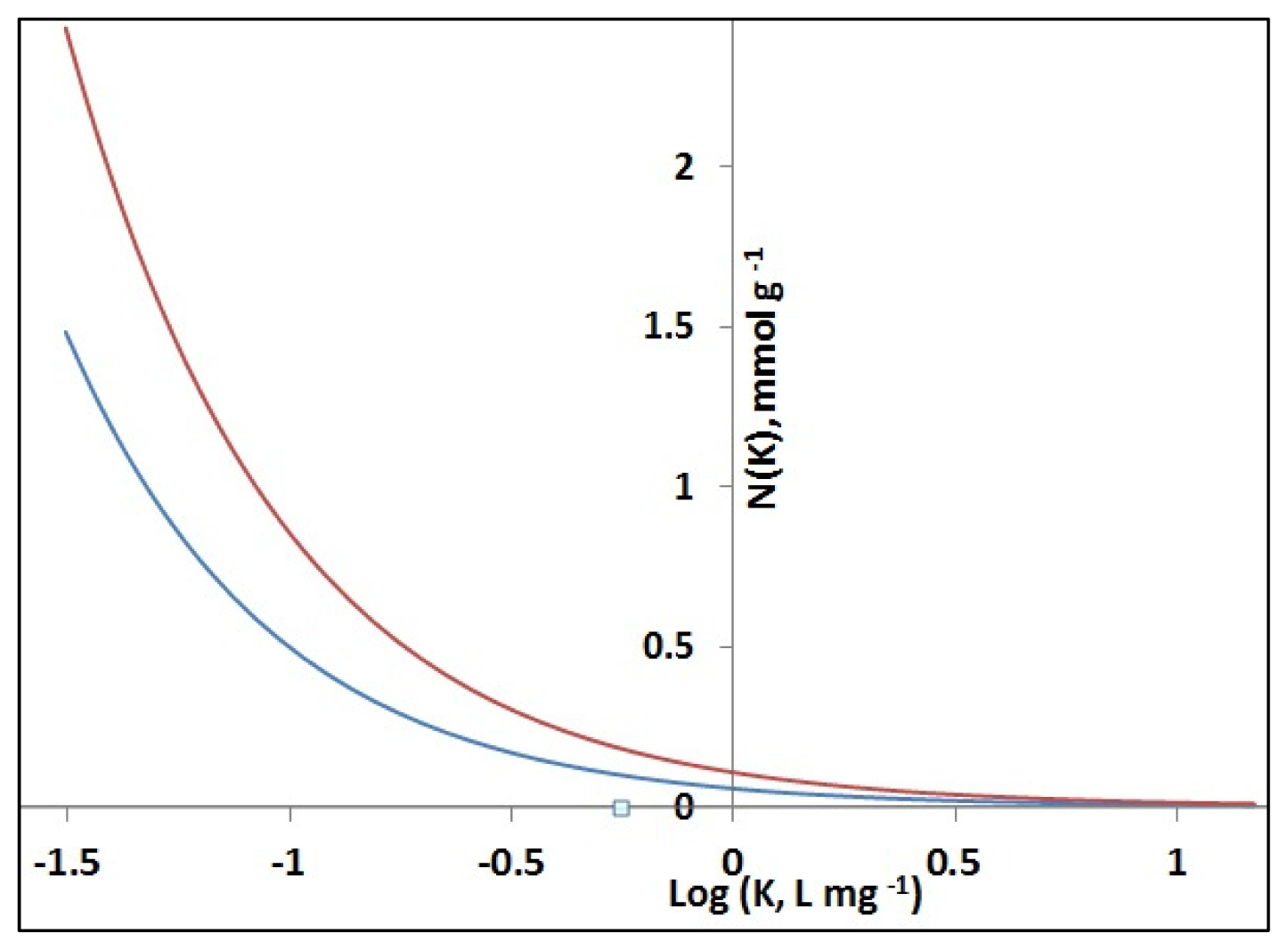

Supplement: Figure S3. — Affinity distributions corresponding to Freundlich Isotherm binding model [31] for MIP 5 on PETA ( ) and corresponding NIP ( ) calculated based on binding parameters (Formula S3). [file ijms-15-01338s3.tif]
